# Erratum for Cazares et al., “A Novel Group of Promiscuous Podophages Infecting Diverse Gammaproteobacteria from River Communities Exhibits Dynamic Intergenus Host Adaptation”

**DOI:** 10.1128/msystems.00426-22

**Published:** 2022-06-13

**Authors:** Daniel Cazares, Adrian Cazares, Wendy Figueroa, Gabriel Guarneros, Robert A. Edwards, Pablo Vinuesa

**Affiliations:** a Centro de Ciencias Genómicas, Universidad Nacional Autónoma de México, Cuernavaca, Morelos, Mexico; b EMBL's European Bioinformatics Institute (EMBL-EBI), Wellcome Genome Campus, Cambridge, United Kingdom; c Wellcome Sanger Institute, Wellcome Genome Campus, Cambridge, United Kingdom; d Department of Biochemistry, University of Cambridge, Cambridge, United Kingdom; e Departamento de Genética y Biología Molecular, CINVESTAV IPN, Mexico City, Mexico; f FAME, Flinders University, Adelaide, SA, Australia

## ERRATUM

Volume 6, no. 1, e00773-20, 2021, https://doi.org/10.1128/mSystems.00773-20. On p. 16, in the first sentence of Acknowledgments, “D.C. gratefully acknowledges the Programa de Doctorado en Ciencias Biomédicas, UNAM” should read “D.C. gratefully acknowledges the Programa de Doctorado en Ciencias Biomédicas, Universidad Nacional Autónoma de México (UNAM).”

