# Supplementary figures and images for: A Novel Group of Promiscuous Podophages Infecting Diverse Gammaproteobacteria from River Communities Exhibits Dynamic Intergenus Host Adaptation
Source: mSystems. 2021 Feb 2;6(1):e00773-20. doi: 10.1128/mSystems.00773-20 (PMC7857530; doi:10.1128/mSystems.00773-20)

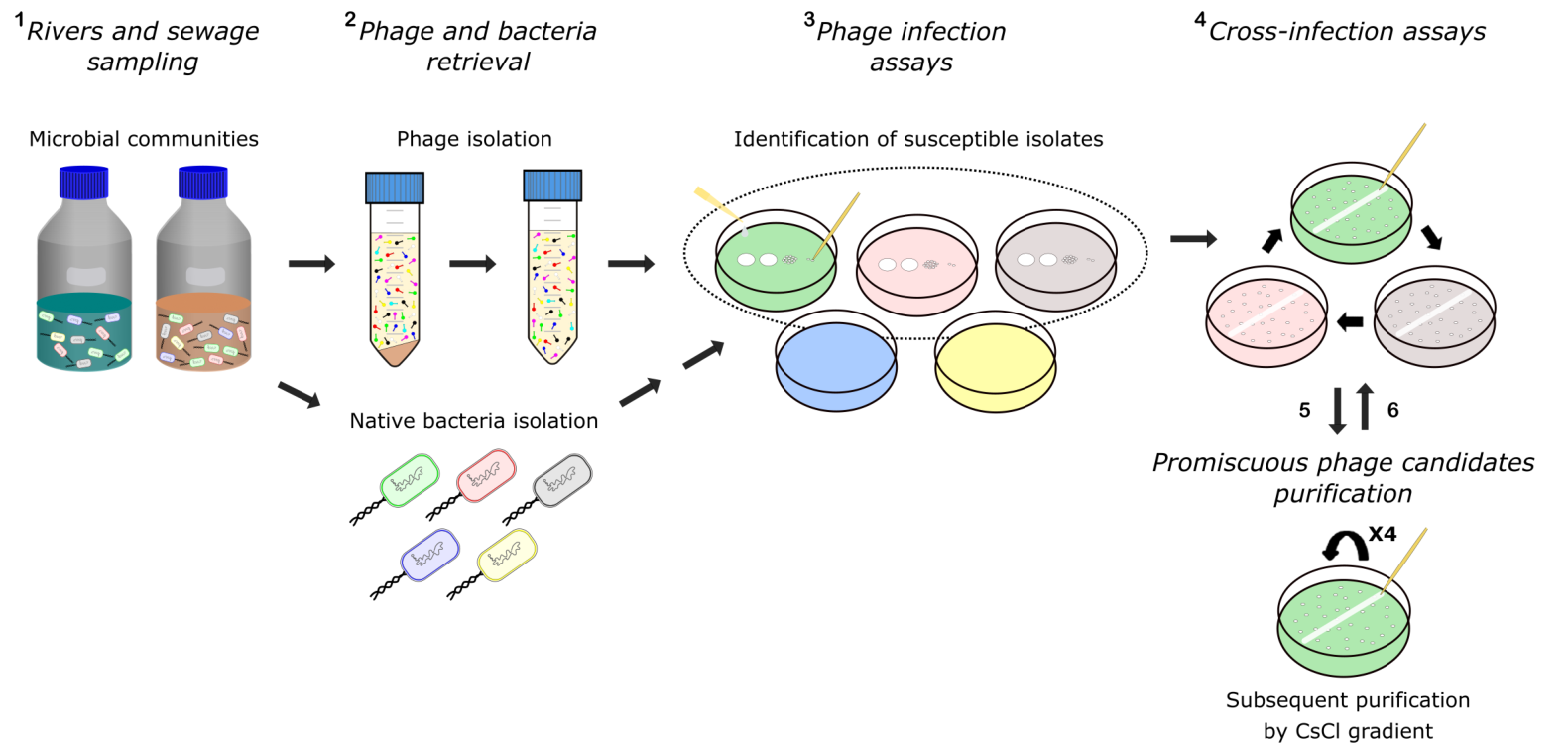

Supplement: FIG S1 [file mSystems.00773-20-sf001.tif]

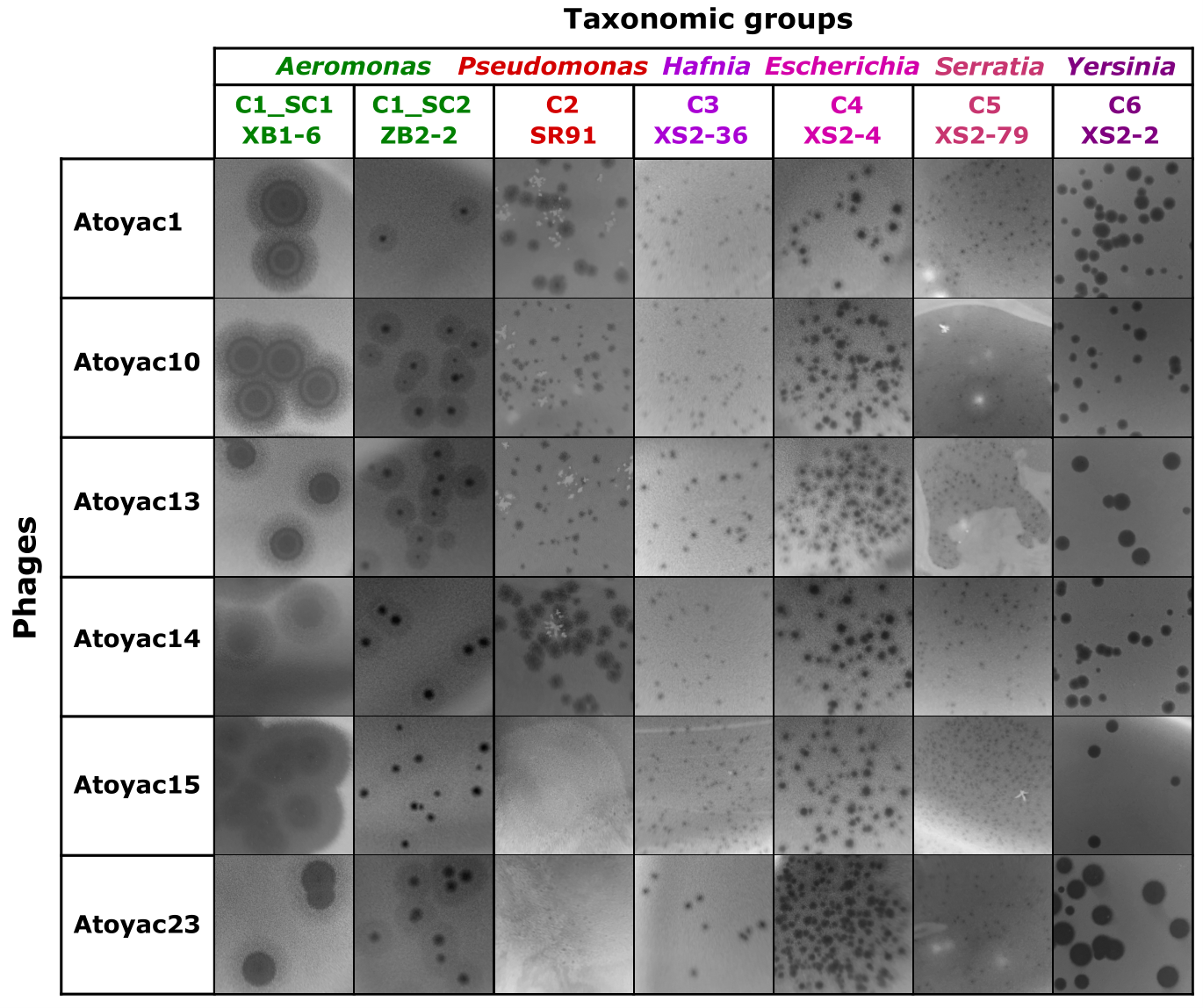

Supplement: FIG S2 [file mSystems.00773-20-sf002.tif]

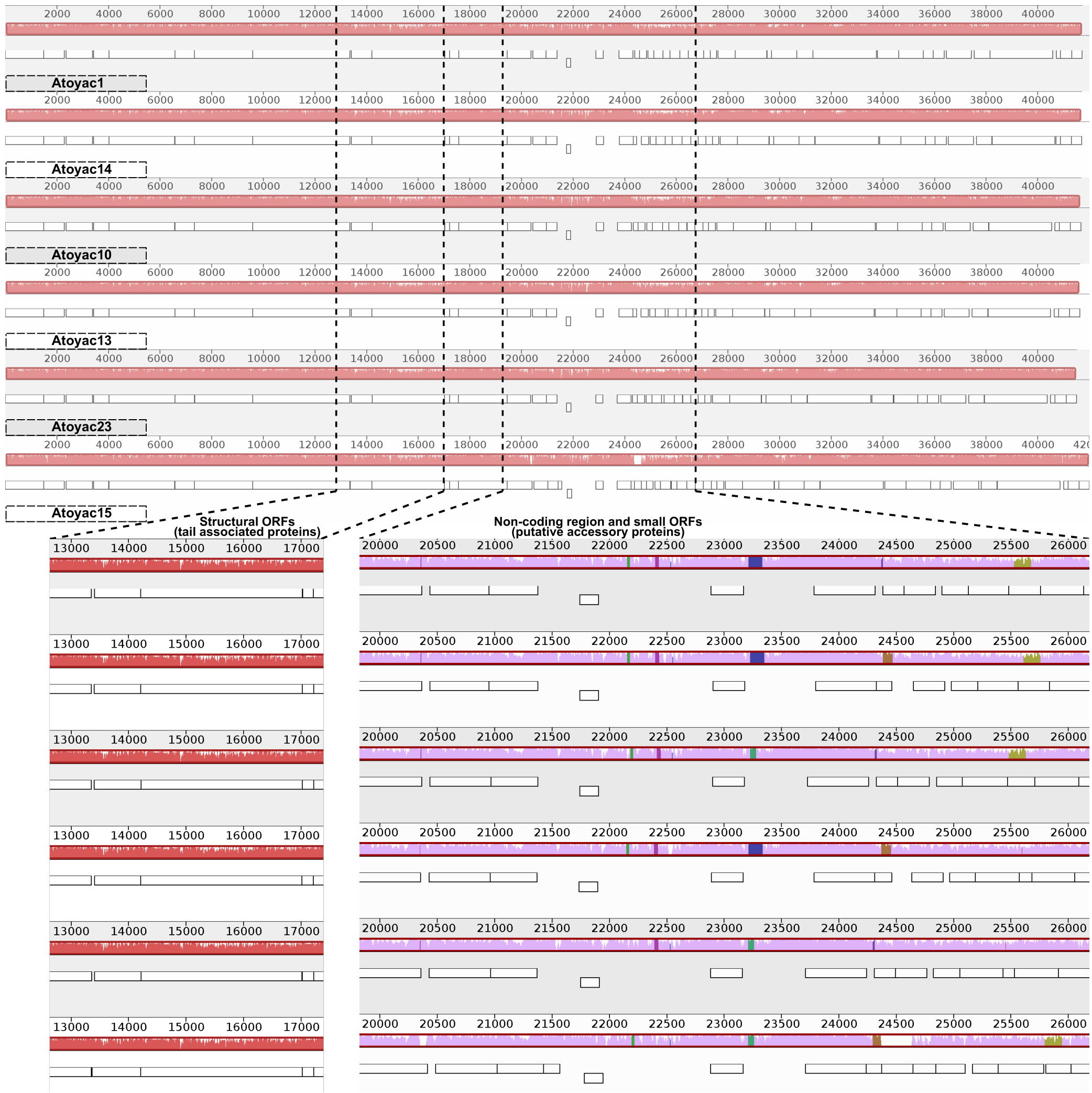

Supplement: FIG S3 [file mSystems.00773-20-sf003.tif]

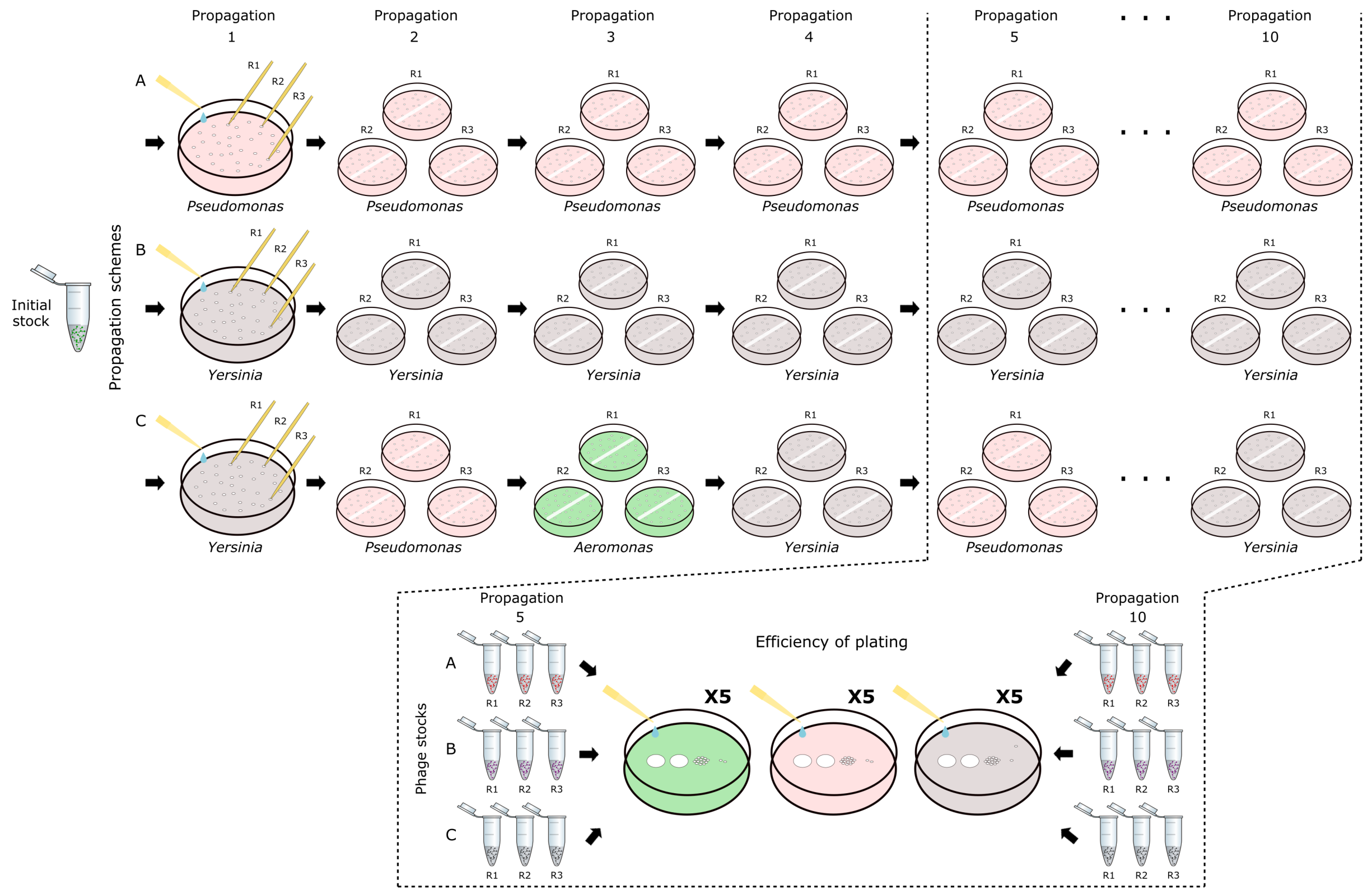

Supplement: FIG S4 [file mSystems.00773-20-sf004.tif]

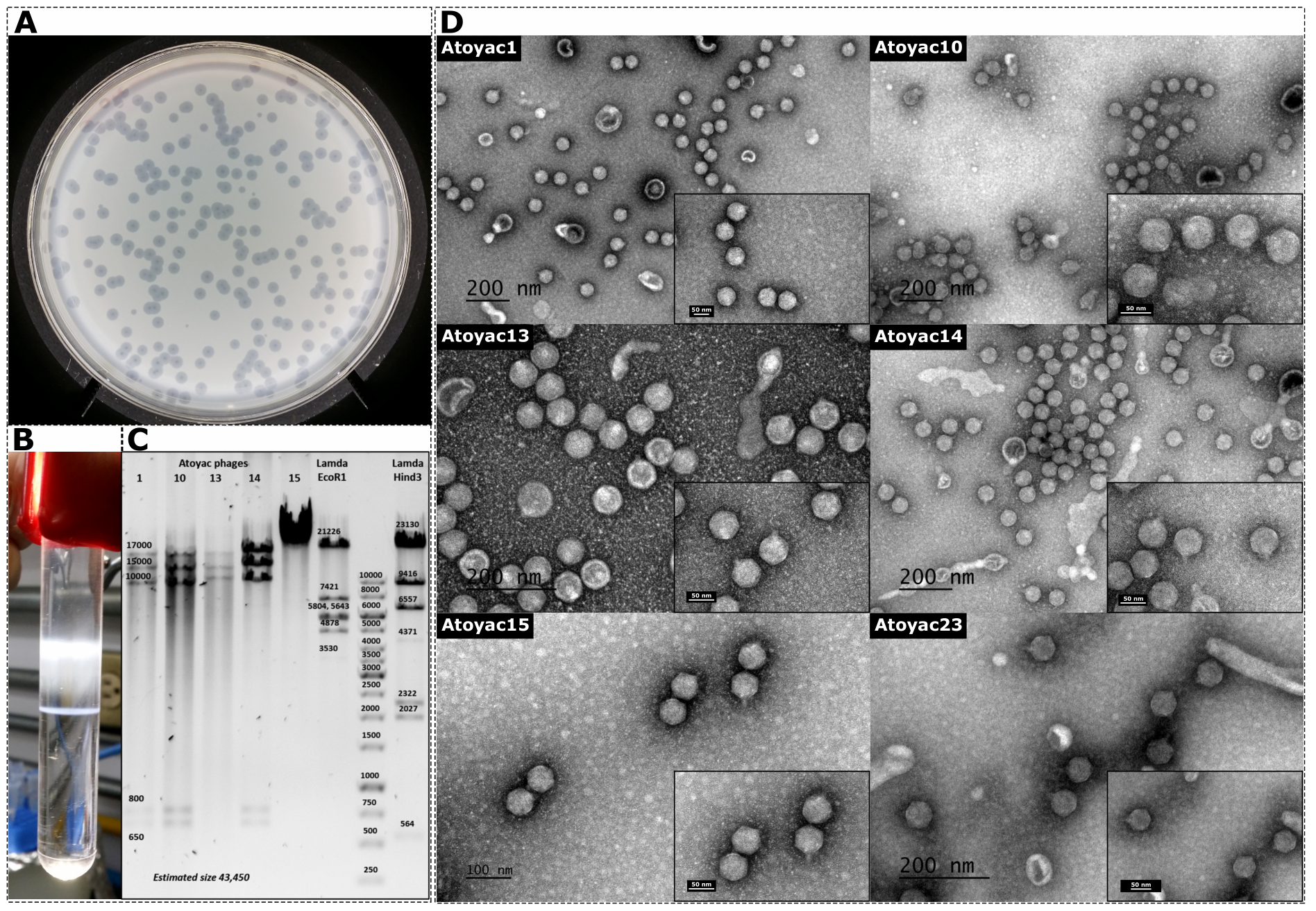

Supplement: FIG S5 [file mSystems.00773-20-sf005.tif]
